# Resistance phenotypes and genomic features of *Mycobacterium seoulense* isolates

**DOI:** 10.3389/fcimb.2025.1553591

**Published:** 2025-04-07

**Authors:** Jin Zhao, Xinli Shen, Lulu Jin, Songjun Ji, Xinling Pan

**Affiliations:** Department of Biomedical Sciences Laboratory, Affiliated Dongyang Hospital of Wenzhou Medical University, Dongyang, Zhejiang, China

**Keywords:** *M. seoulense*, whole genome sequencing, virulence factor, drug susceptibility, intracellular survival

## Abstract

**Background:**

*Mycobacterium seoulense* (*M. seoulense*) is an emerging pathogen increasingly associated with infections; however, its resistance phenotypes and genomic characteristics remain largely unknown.

**Methods:**

Seven *M. seoulense* isolates were collected from clinical samples. Drug susceptibility testing was conducted using Sensititre™ SLOMYCO2 susceptibility plates. Whole genome sequencing and supporting bioinformatics analyses were performed to analyze the genomic features.

**Results:**

All *M. seoulense* isolates (n=7) exhibited growth on 7H10 agar medium containing thiophenecarboxylic acid hydrazide or p-Nitrobenzoic acid, with marked diversity in growth rates in liquid culture. All strains exhibited high minimum inhibitor concentrations (MICs) for minocycline (>8 μg/mL), doxycycline (>8 μg/mL), and amikacin (16-32 μg/mL). The MICs for linezolid, rifabutin, moxifloxacin, ciprofloxacin, streptomycin, clarithromycin, and rifampicin varied among the isolates. High levels of genomic diversity were noted among these strains concerning genome-called single nucleotide polymorphisms and average nucleotide identity. In total, 4,282 genes were shared across all genomes, while 315 unique genes were restricted to one strain. Comparative genomic analysis identified two unique virulence genes encoding a catalase enzyme and a protein involved in capsule biosynthesis and transport. Additionally, all *M. seoulense* strains demonstrated the ability to survive within macrophages.

**Conclusion:**

The clinical *M. seoulense* isolates analyzed in this study exhibited varying levels of antibiotic susceptibility, suggesting the potential need for susceptibility testing to guide clinical treatment. Genomic features of these isolates indicated that they are likely pathogenic non-tuberculous mycobacterium, highlighting a need for closer epidemiological monitoring.

## Introduction

Most non-tuberculous mycobacteria (NTM) are widely distributed in the environment, including dust and water sources. Although typically nonpathogenic, they can cause infections in immunocompetent individuals, resulting in pulmonary, soft tissue, and lymphatic infections (Nguyen et al., 2024; Sun et al., 2024). Among immunosuppressed patients, NTM can also cause disseminated infections (Kitazawa et al., 2024; Nithirungruang et al., 2024), posing an immense therapeutic challenge to those with preexisting diseases. With the rapid development of molecular detecting techniques, over 200 species of NTM have been identified to date (https://lpsn.dsmz.de/genus/mycobacterium) (Armstrong et al., 2023), many of which have been implicated in clinical infections. Several treatment guidelines have been proposed for NTM infections (Haworth et al., 2017; Kurz et al., 2020), but there remains limited evidence regarding the most appropriate therapeutic regimens when combatting infections caused by less common NTM species (Lange et al., 2022). Therefore, phenotypic and genomic investigations are warranted for those novel NTM species with rising clinical prevalence (Zhang et al., 2013).

*Mycobacterium seoulense* (*M. seoulense*), which was first reported in Korea in 2007 (Mun et al., 2007), has also been found to cause pulmonary infection in Japan and China (Zhao et al., 2022; Nakamoto et al., 2024). Four cases of *M. seoulense* infection were identified in our previous study through radiological examination. However, anti-mycobacterial treatment was not initiated due to limited clinical experience, a lack of public data on *M seoulense* infections, and patients’ reluctance to receive antibiotic therapy. Only the case from Japan received antibiotic therapy, and had a optimal outcome. While the growth features and biochemical features of this NTM have been reported (Mun et al., 2007), the resistance phenotype for *M. seoulense* remains unknown. Like most NTM, the addition of thiophene carboxylic acid hydrazide (TCH) and p-nitrobenzoic acid (PNB) to the culture medium has no impact on the growth of *M. seoulense*, potentially providing a means of differentiating it from *M*. *nebraskense*, a biochemically similar species. Although two genomes for *M. seoulense* are available (https://www.ncbi.nlm.nih.gov/datasets/genome/?taxon=386911), no genomic analysis has been conducted, leaving some uncertainty regarding the relevant virulence factors and core genes for this species. Therefore, in this study, seven strains isolated from clinical samples in our hospital were sequenced, and drug susceptibility test was performed to provide clinicians with insights to help guide the management of patients infected by *M. seoulense*.

## Methods

### Bacterial culture

Strains were recovered from -80°C by subculturing them in Middlebrook 7H9 broth medium (BD Bioscience, USA) containing 10% ADC broth enrichment medium (Shanghai Yiyan Bio-technology Co. Ltd, Shanghai, China) and 0.05% Tween-80. Isolates were incubated at 37°C until reaching an optical density at 600 nm (OD600) of 0.6-1.0 as measured with an Implen (Germany) instrument. A shaking culture was then performed at 37°C and 250 rpm, and growth curves were obtained by testing the OD600 every three days before day 21 and every six days after day 21. In addition, the medium was imaged after an 18-day incubation.

To assess the morphology of these isolates on the Middlebrook 7H10 agar medium, cultures with an OD600 between 0.6 and 1.0 were diluted to an OD600 of 0.02 using a complete 7H9 medium, and 5 µL of the culture was spotted onto a 7H10 complete agar plate supplemented with 10% glycerol. The 7H10 agar plates were supplemented with 5 μg/mL of TCH or 500 μg/mL of PNB. The plates were incubated in an incubator at 37°C and imaged until the colonies had formed after 21 days.

### Drug susceptibility test

Bacterial cultures with an OD600 between 0.6 and 1.0 were obtained as described above, followed by dilution with phosphate-buffered saline (PBS) to a final OD600 of 0.2 (about 0.5 McFarland) (Penuelas-Urquides et al., 2013). The bacterial suspension was then diluted 100-fold using Mueller-Hinton medium, and 100 μL was transferred into the Sensititre™ SLOMYCO2 drug susceptibility test plates (Thermo Fisher, USA). These plates were sealed with parafilm and cultured in an incubator at 37°C. Readings were taken after 7 days, with the minimum inhibitory concentration defined as the lowest drug concentration at which no visible bacterial sediment was observed at the bottom of the well. Two replicate wells were established for this drug susceptibility assay*. M. intracellulare* ATCC 13950 was used as the reference strain.

### Genomic DNA extraction and next-generation sequencing

Bacteria in the early exponential phase were harvested via centrifugation, and cell pellets were used to extract genomic DNA based on the provided instructions (Tiangen, Beijing, China). High-quality DNA was used to prepare a library with a NEXTFLEX Rapid DNA-Seq Kit (Revvity, MA, USA). Sequencing was performed on a NovaSeq™ X Plus (Illumina, CA, USA), and raw data was analyzed to remove low-quality reads using the fastp software (Chen et al., 2018). The high-quality reads were assembled with Unicycler software (Wick et al., 2017), and the sequences were uploaded to the NCBI database (PRJNA1196008). Genes were annotated using Prokka (Seemann, 2014), and pan-genome analyses were conducted using Roary (Page et al., 2015).

### Genomic analysis of the assembled sequences

*M. seoulense* strain JCM16018 was the reference genome (Accession No: GCA_010731595.1) for calling core single nucleotide polymorphisms (coreSNPs) of clinical isolates using the Snippy software as described previously (Seemann, 2015). Following the removal of SNPs in recombinant regions using Gubbins (Croucher et al., 2015), the coreSNP distribution matrix was used to construct a Phylogenic tree with IQ-TREE (Nguyen et al., 2015). Genomic alignment was performed via BLAST and visualized in the form of rings with BRIG (Alikhan et al., 2011). The identity percentages between any two strains were evaluated based on average nucleotide identity (ANI) (Yoon et al., 2017).

### Third-generation sequencing

Genomic DNA was fragmented into sequences ~10 kb in length, after which an SMRT Bell library was constructed with an SMRT Bell Prep Kit 3.0 (Pacific Biosciences, CA). The raw data was sequenced using the PacBio Sequel IIe sequencing platform. Genomic assembly was performed with the Unicycler software, and the results were uploaded to the NCBI database (PRJNA1195676). The whole genome was aligned to the JCM16018 strain, and the arranged genomes were analyzed using Mauve (Darling et al., 2004).

### Analyses of intracellular survival in macrophages

THP-1 cells were purchased from the China Center for Type Culture Collection (Shanghai, China) and cultured in RPMI 1640 medium (Gibco, USA) containing 10% fetal bovine serum (Vivacell, China). Cells were seeded into 24-well plates (1 x 10^5^/well) and differentiated into macrophages via treatment for 24 h with 60 ng/mL phorbol 12-myristate 13-acetate. Bacterial cultures with an OD600 between 0.6 and 1.0 were harvested by centrifugation (2,000 rpm, 5 min). After washing thrice with PBS, the bacterial pellet was resuspended as a single-cell suspension in RPMI 1640 medium via repeated pipetting. The concentration was adjusted into an OD600 of 0.6 (equivalent to 1×10^8^/mL) and co-cultured with the macrophage at a multiplicity of infection of 10. After three hours of incubation, the extracellular bacteria were washed away twice with PBS and killed by adding 30 µg/mL gentamycin for one hour. The infected cells were incubated in a complete RPMI 1640 medium supplemented with 5 µg/mL gentamycin in a 37°C 5% CO_2_ incubator. After incubation for 24 h, the cells were lysed with sterile deionized water and serially diluted with PBS. The diluted lysed cell solutions were spread on the 7H10 agar medium plates with 10% glycerol. After incubation for 20 days, the colony-forming units (CFUs) on these plates were counted. The survival rate was calculated as the ratio of CFU obtained after infection for 24 h to the initial CFU obtained after infection. This experiment was repeated three times.

### Statistical analysis

The survival rates of *M. seoulense* within macrophages were compared to that of *M. intracellulare* ATCC13950 strain using a *t*-test in GraphPad Prism (version 9.3.1). Statistical significance was defined as *, *P*<0.05; **, *P*<0.01.

## Results

### Biological features of *M. seoulense* in culture

**Figure 1:**
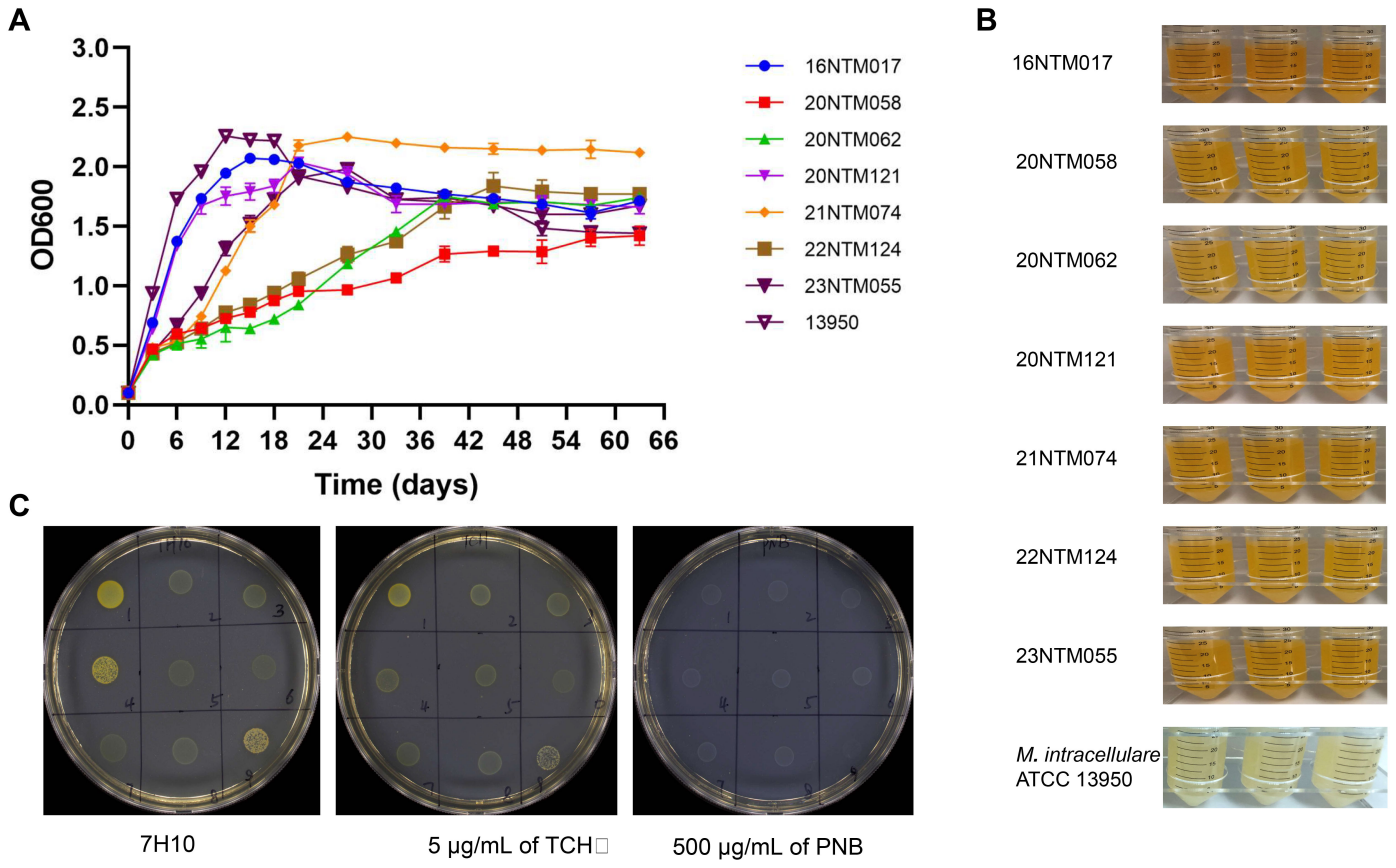
The growth features of *Mycobacterium seoulense* in culture medium. **(A)** The growth curve in the 7H9 Middlebrook medium with 10% ADC and 0.05% Tween 80; **(B)** The color of 7H9 medium liquid medium; **(C)** The morphology of *Mycobacterium seoulense* on the 7H10 agar medium with 10% glycerol. 1, 16NTM017; 2, 20NTM058; 3, 20NTM062; 4, 20NTM121; 5, 21NTM074; 6, 21NTM160 (*M. colombiense*); 7, 22NTM124; 8, 23NTM055; 9, ATCC13950.

A total of seven *M. seoulense* strains were isolated from six patients (**Supplementary Table 1**), including four previously described cases. The two additional cases in this study had *M. seoulense* in their bronchoalveolar lavage fluid specimens. In the 7H9 broth medium, these clinical isolates exhibited different growth rates (**Figure 1A**). Specifically, the 16NTM017 and 20NTM121 isolates grew more rapidly than the 23NTM055 and 21NTM074 isolates, with these four strains entering the stationary phase after about 21 days, similar to *M. intracellulare* ATCC 13950. The 20NTM058, 20NTM062, and 22NTM124 strains grew gradually and entered the stationary phase until 40 days. The color of the liquid culture medium for all *M. seoulense* isolates was brown-yellow, while that for *M. intracellular* was opalescent (**Figure 1B**).

On solid medium, all isolates were able to grow on the 7H10 complete medium with or without TCH or PNB (**Figure 1C**). However, the clones grown on PNB were white, whereas those grown on other mediums were yellow. Moreover, strains 16NTM017 and 20NTM121 exhibited a darker color compared to other strains.

### Drug susceptibility test of *M. seoulense* isolates

All *M. seoulense* strains exhibited high MICs for Amikacin, ranging from 16-32 μg/mL (**Table 1**). For doxycycline and minocycline, all the strains exhibited MIC levels higher than the upper limit of detection. These clinical isolates presented varying levels of susceptibility to moxifloxacin, ciprofloxacin, streptomycin, and clarithromycin, as indicated by at least four MICs for these seven strains.

**Table 1:** Distribution of MICs for *M. seoulense* strains.

Strain name	MIC (μg/mL)										
	DOX	LZD	RFB	AMI	MXF	CFZ	CIP	STR	CLA	MIN	RIF
16NTM017	>8	4	4	16	0.25	1	1	2	0.25	>8	1
20NTM058	>8	8	0.25	16	2	0.5	>8	16	0.5	>8	2
20NTM062	>8	32	1	32	4	1	>8	>32	2	>8	4
20NTM121	>8	16	>4	16	2	1	>8	16	1	>8	0.25
21NTM074	>8	16	0.12	16	1	1	8	16	2	>8	0.25
22NTM124	>8	16	0.5	16	1	1	4	8	1	>8	2
23NTM055	>8	16	0.25	32	1	1	4	8	2	>8	2
ATCC13950	>8	8	0.25	4	1	1	2	4	1	4	0.25

DOX, doxycycline; LZD, linezolid; RFB, rifabutin; AMI, amikacin; MXF, moxifloxacin; CFZ, clofazimine; CIP, ciprofloxacin; STR, streptomycin; CLA, clarithromycin; MIN, minocycline; RIF, rifampicin.

### Phylogenetic analyses of *M. seoulense* isolates

**Figure 2:**
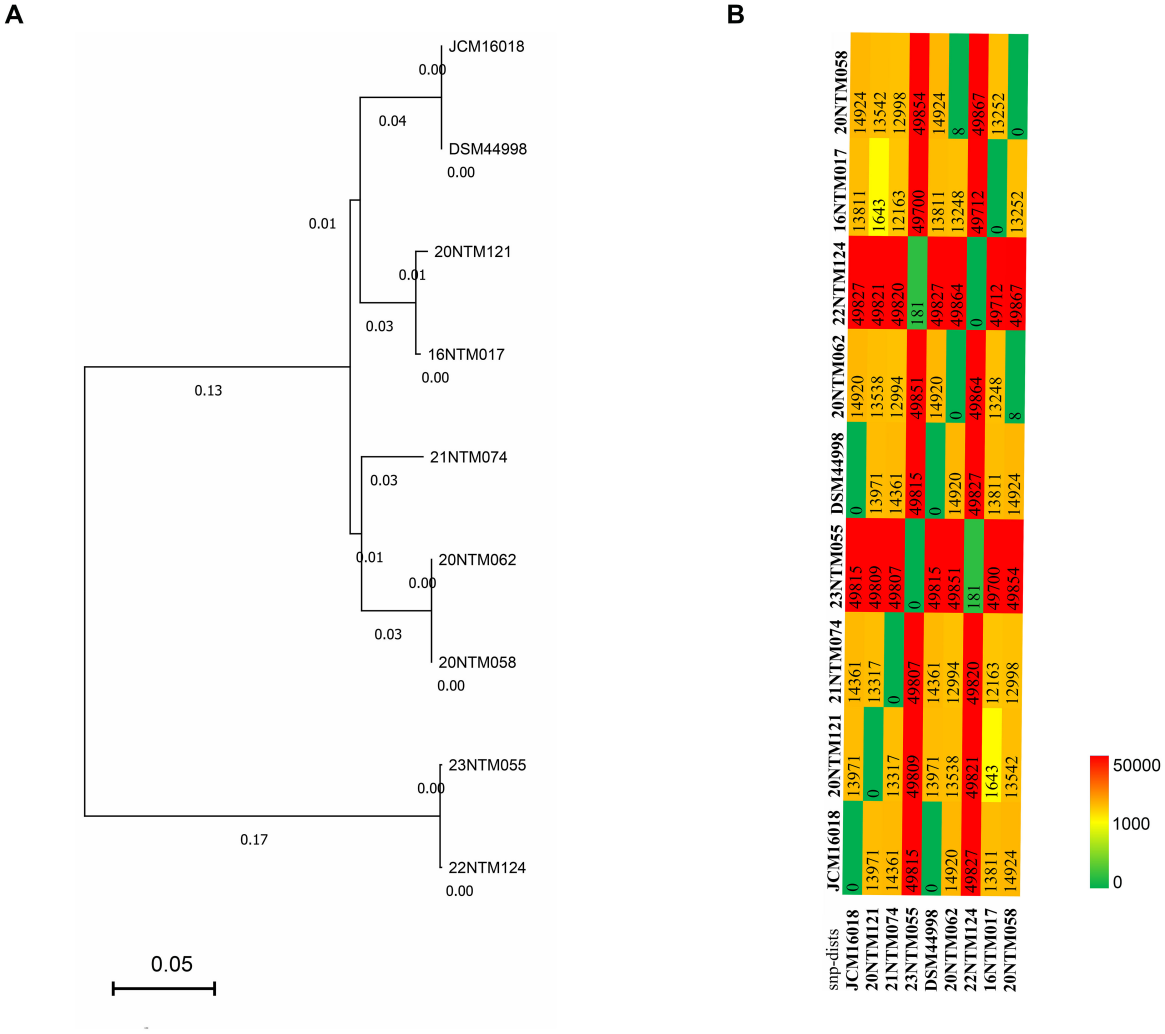
The phylogenetic analysis of *Mycobacterium seoulense* strains. **(A)** phylogenetic tree based on the coreSNPs; **(B)** distribution matrix of coreSNPs among *Mycobacterium seoulense* strains.

Based on the coreSNPs detected in individual strains, an established phylogenetic tree revealed the clustering of the JCM16017 and DSM 44998 strains (**Figure 2A**), with an average nucleotide identity of 99.99% (**Supplementary Figure 1**). The two strains derived from the same case (20NTM062 and 20NTM058) were also clustered with ANI of 99.98%. Two strains that were isolated from different cases were close to each other in the phylogenetic tree. The differing SNP distributions among the analyzed strains provided support for the phylogenetic tree results (**Figure 2B**). For the clustered strains, the strains derived from the same case exhibited eight coreSNPs that differed from one another, while those from different cases exhibited 181-1643 differing coreSNPs. In addition, 22NTM124 and 23NTM055 were distant from the other strains (**Figure 2A**), with approximately 50,000 coreSNPs differing from the other strains (**Figure 2B**). The diversity of coreSNPs was similar to the ANI distributions between any two strains (**Supplementary Figure 1**).

### Pan-genome analyses of *M. seoulense* isolates

**Figure 3:**
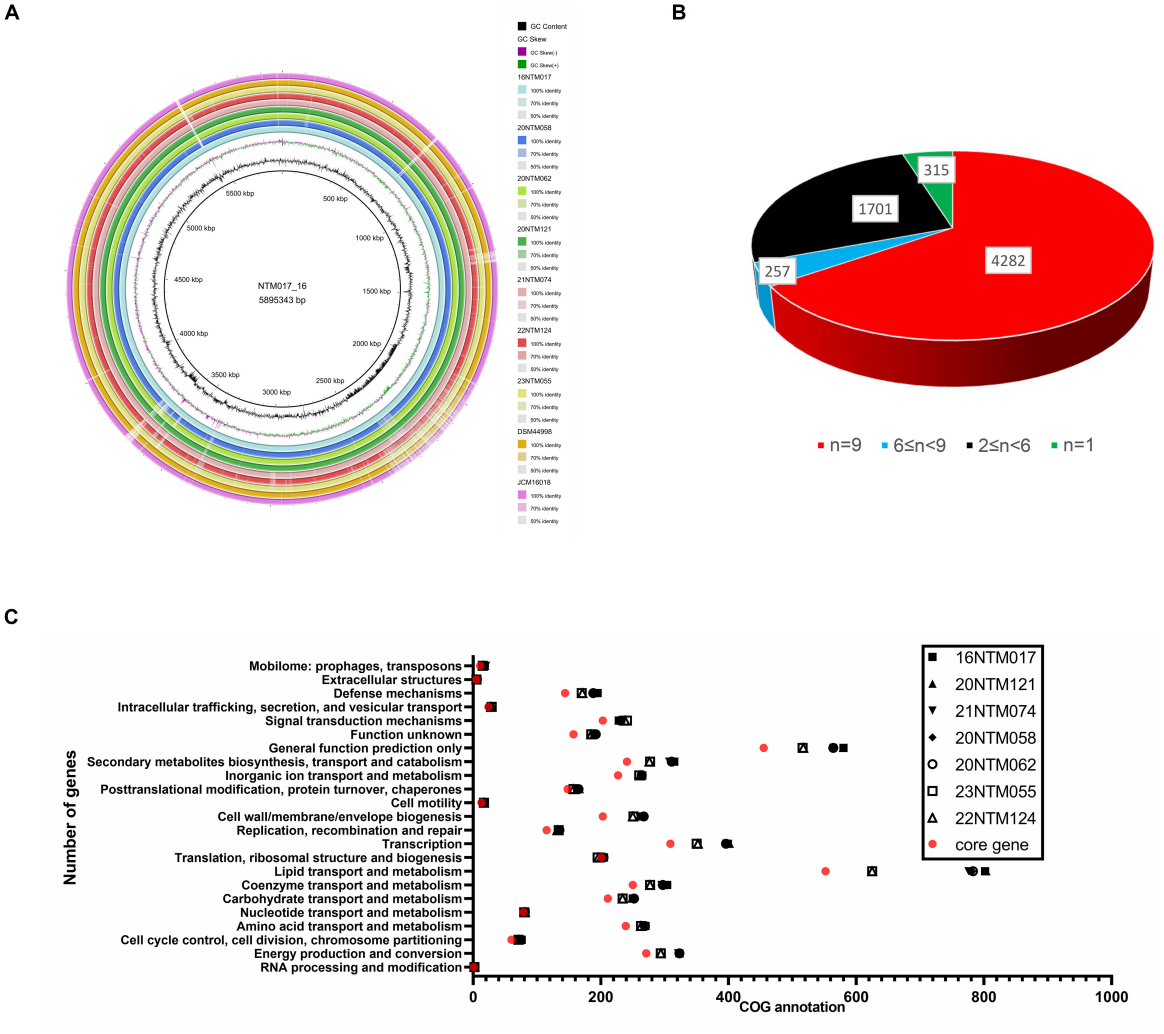
The pan-genome analysis of *Mycobacterium seoulense* strains. **(A)** Genomic alignment by circular ring; **(B)** A summary of genes in the pan-genome analysis; **(C)** The number of genes with COG annotation of genes in the pan-genome analysis.

In order to establish the core genes of *M. seoulense*, nine isolates, including our clinical isolates and reference strains, were subjected to alignment and pan-genome analyses. Relative to the 16NTM017 strain, diversity hot spots were observed across the genome, including from 1965.5-2391 kb and 3600-3700 kb (**Figure 3A**). These regions exhibited a higher percentage of GC content than other regions. For the genes in these strains, 4,282 conserved genes were present in all genomes, while 315 unique genes were present in a single strain (**Figure 3B**, **Supplementary Table 2**). There were 552 genes associated with lipid transport and metabolism, accounting for 15.4% of the 3,595 genes with available COG annotation. This category was associated with the most diversity in terms of the number of related genes among these clinical isolates, while the numbers of genes associated with “cell motility”, “extracellular structure”, and “intracellular trafficking, secretion, and vesicular transport” were largely conserved (**Figure 3C**).

### Genomic analyses of *M. seoulense*

**Figure 4:**
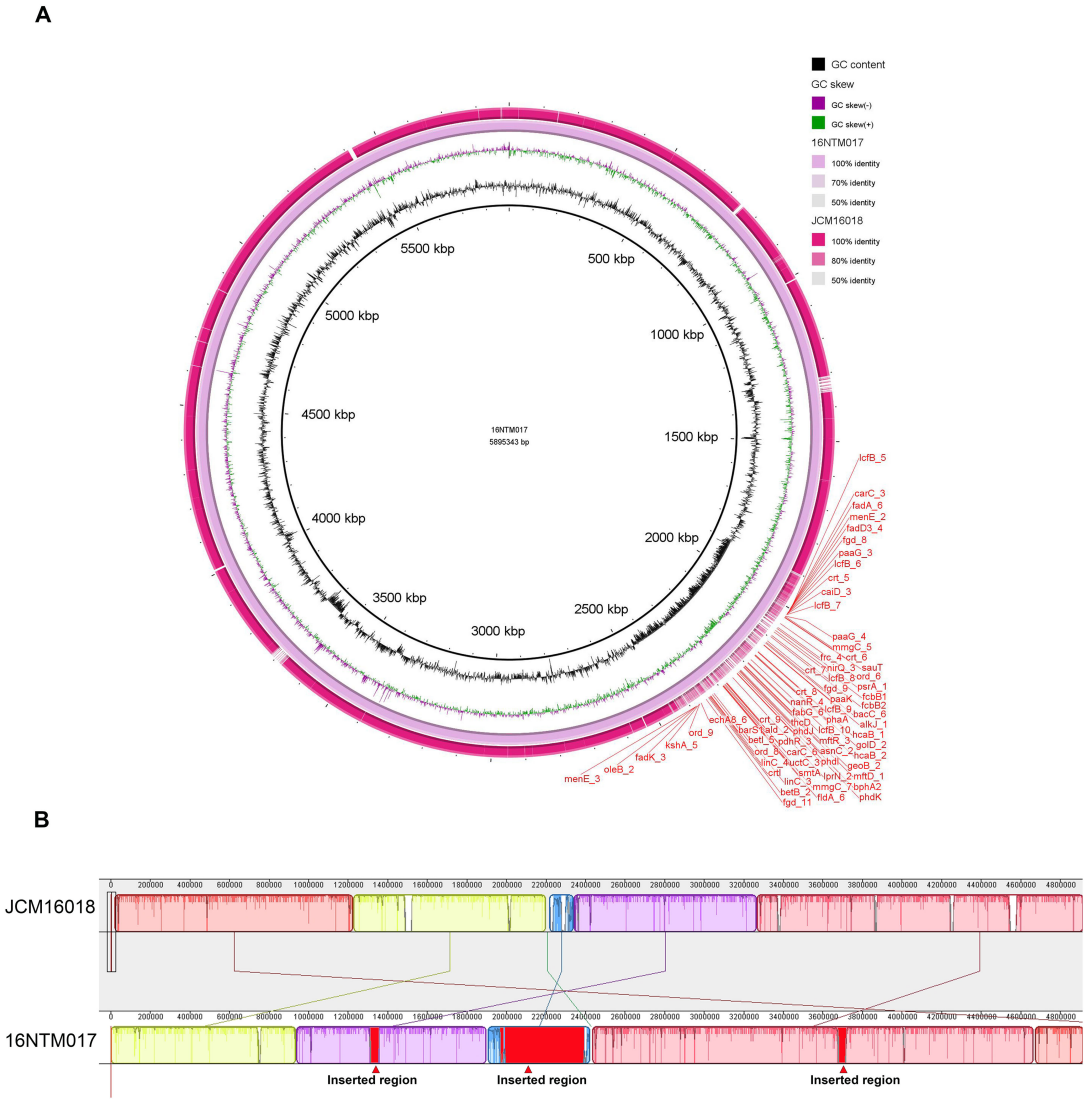
The genomic alignment of 16NTM017 and JCM16018 based on the assembled whole genome sequence. **(A)** The global view of genomic alignment; **(B)** the rearrangement of genomic regions between the aligned genomes. The red regions indicated for inserted regions in 16NTM017 comparing to JCM16018.

In order to comprehensively analyze the genomic features of 16NTM017, assembled genomic data was obtained via third-generation sequencing. Relative to strain JCM16018, 16NTM017 exhibited an ANI of 99.03% (**Figure 4A**), with several long insertion sequences and one rearranged region in the genome (**Figure 4B**). The longest inserted sequence corresponded to position 1965.5 kb to 2391 kb, encoding 434 CDS.

### Virulence factors in *M. seoulense*

**Figure 5:**
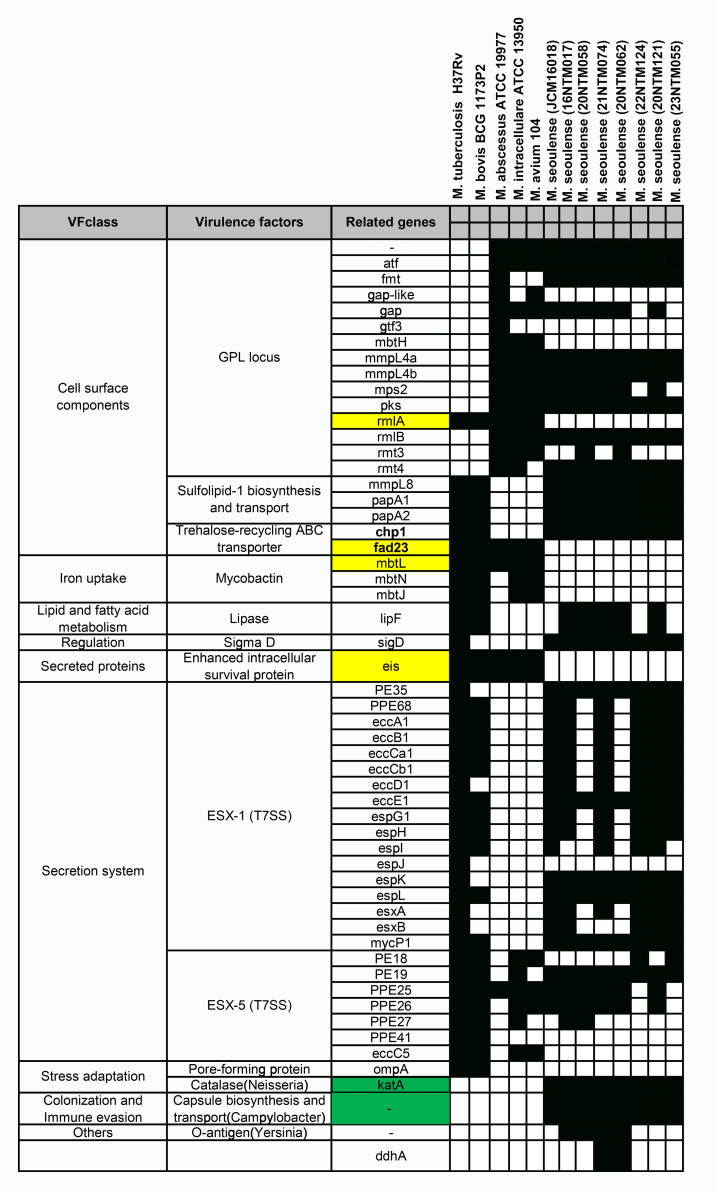
The virulence factor-related genes for *Mycobacterium seoulense*. The genes with yellow background were specifically absent in *Mycobacterium seoulense* strains comparing to other mycobacterial species; The genes with green background were uniquely present in the *Mycobacterium seoulense* strains comparing to other mycobacterial species.

When compared with three reference strains (*Mycobacterium tuberculosis*, *Mycobacterium bovis BCG* strain, and *Mycobacterium avium* complex (MAC)), the reference *M. seoulense* strain and our clinical isolates exhibited a range of common virulence factors (**Figure 5**). Specifically, the *M. seoulense* strains were found to harbor two unique genes encoding a catalase enzyme and a protein involved in capsule biosynthesis and transport. In addition, the *M. seoulense* strains were found to lack *rmlA*, *mbtL*, *fad23*, and *eis*, which are associated with the GPL locus, a trehalose-recycling ABC transporter, mycobactin, and the enhanced intracellular survival protein. The distribution of several genes also varied among clinical isolates (**Supplementary Table 3**).

### Analyses of intracellular survival in macrophages

**Figure 6:**
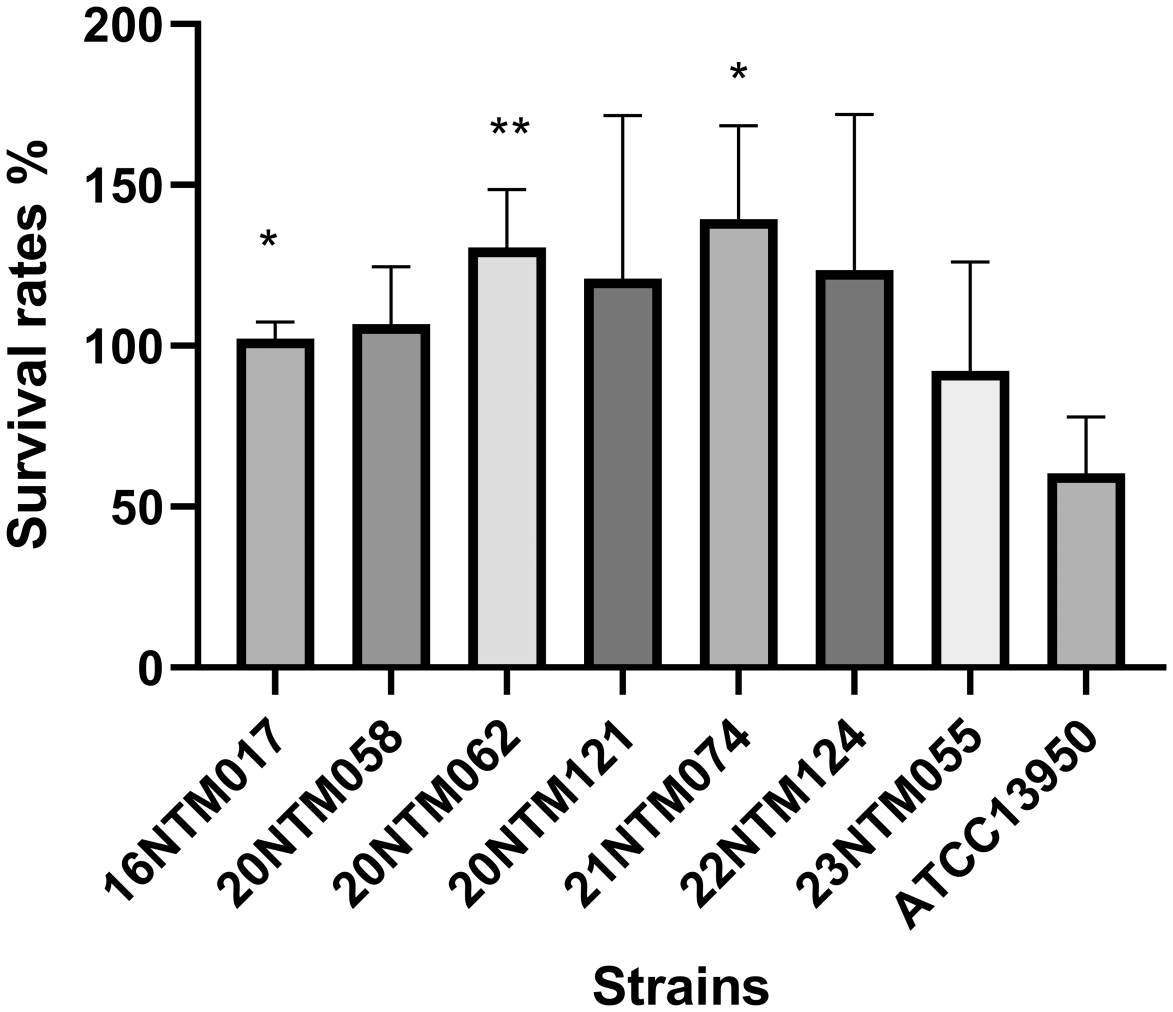
The intracellular survival of *Mycobacterium seoulense* strains within the macrophage. The survival rate was defined as the ratio of CFU after 24 hours infection to CFU at the time of infection. *, P<0.05; **, P<0.01.

The *M. intraellulare* reference strain ATCC13950 exhibited a survival rate of ~60% in macrophages for 24 h (**Figure 6**). Relative to *M. intracellulare*, the clinical *M. seoulense* isolates demonstrated enhanced intracellular survival in macrophages, albeit with variations among these strains.

## Discussion

In this study, whole genome sequencing was performed to characterize this novel mycobacterial species and to identify virulence factors related to pathogenicity. These efforts provided an opportunity to analyze the genomic features of clinical *M. seoulense* isolates. Compared to the commonly isolated NTM species, such as the *Mycobacterium avium* complex, *M. seoulense* strains exhibited a yellow pigmentation in culture, indicating carotenoid production (Robledo et al., 2011; Tran et al., 2020). In mycobacteria, the carotenoid production is used mainly for taxonomic identification. However, in other bacterial species, carotenoids possess antioxidant, anti-inflammatory, anti-cancer, and antimicrobial properties (Tran et al., 2020). Although most NTM can be isolated from living environments and medical devices, *M. seoulense* has not been reported in the soil or water (Abbas et al., 2024; Zhang et al., 2024). One explanation is that it was overlooked in epidemiological studies, as *M. seoulense* infections tend to be less common in clinical practice relative to other NTM species (Zhao et al., 2022; Nakamoto et al., 2024). Owing to the absence of data regarding the recommended threshold of SNPs in defining clonal transmission in this species, evidence of transmission between these cases could not be obtained based on the available epidemiological data. Based on the SNPs that differed between the strains isolated from the same patient (8 SNPs), the genomic diversity among clonal *M. seoulense* strains may be greater than for other species belonging to the MAC (Namkoong et al., 2021; Wetzstein et al., 2024). Different drug susceptibility phenotypes for several antibiotic agents were indicated for high heterogeneity of these two strains. Interestingly, two *M. seoulense* strains with available genomes (DSMD44998 and JCM16018) shared highly similar genomes despite their submission by different individuals. Moreover, their identity percentages were higher than those from the same case in this study, indicating the possibility of a common source. This finding warrants further study, as these microbes were isolated from different countries, potentially indicating interregional transmission. Unfortunately, the possible epidemiological connections for these two strains could not be established based on the information submitted to the database.

The MICs for *M. seoulense* were first reported in this study, but the resistance phenotypes could not be established owing to limited data on these MICs and recommended breakpoints (Brown-Elliott and Woods, 2019). Based on its growth feature, *M. seoulense* is classified as a slow NTM (Mun et al., 2007). When using the breakpoints for slow-growth NTM species other than for MAC or *M. kansasii* used in a previous study (Liu et al., 2021)*, M. seoulense* strains could be not efficiently inhibited by doxycycline, minocycline, or ciprofloxacin. Based on susceptibility results, a multi-drug regimen containing clofazimine or clarithromycin in combination with rifampicin and moxifloxacin may be effective for *M. seoulense* infections, offering a potential alternative drug regimen in clinical practice (Nakamoto et al., 2024). In cases of suboptimal treatment response, drug susceptibility test for rifampicin and clarithromycin may be necessary, as the MICs vary among isolates. However, further research is required to establish resistance breakpoints, evaluate the efficacy of different drug regimens in clinical settings, and assess treatment outcomes for *M. seoulense* infections (Huh et al., 2019; Kwon et al., 2019).

There was substantial diversity among the analyzed *M. seoulense* genomes in this study based on identity and annotated genes. Despite two strains isolated from the same region, a higher level of diversity was noted among strains. Pan-genomic analyses allowed us to focus on conserved genes in molecular diagnosis and pathogenicity investigations while assessing accessory genes helps to study intra-species diversity (Zekic et al., 2018; Zakham et al., 2021). Further research revealed that the diversity among these clinical strains was related to the number of genes, especially for those annotated in the category “lipid transport and metabolism”. Variations in lipid metabolism-related genes among clinical isolates are also observed in different lineages of *Mycobacterium tuberculosis*, contributing to differences in virulence and transmissibility (O’Neill et al., 2015; Moopanar and Mvubu, 2020). In addition, pathogenic mycobacteria exhibit a contraction in genes related to lipid and secondary metabolite biosynthesis and metabolism (Zhu et al., 2023). Given that lipids are a critical source of energy supporting bacterial survival under nutrient-limiting conditions, the diversity of lipid-related genes may play a significant role in mycobacterial virulence and could have implications for drug development (Bailo et al., 2015). The inconsistent number of transport-associated genes may have contributed to the diverse pathogenicity in the cell infection model and the varying levels of drug resistance (Samuels et al., 2022). In contrast, the genes associated with nucleotide transport and metabolism were more conserved than those related to carbohydrate, amino acid, and lipid transport and metabolism. However, further investigation will be necessary owing to the limited data on the genomic analyses of metabolic activity for NTM (Karmakar and Sur, 2024).

The distribution of virulence factor-related genes in *M. seoulense* may provide insight into the mechanisms that may be responsible for infection, helping to guide the selection of molecular targets for drug design efforts. Mycobactin is important for supporting mycobacterial survival under iron-stress conditions in host macrophages through siderophore formation (Luo et al., 2005; Shyam et al., 2021; Shankar and Akhter, 2024). There are 14 genes in the *mbt* gene clusters that are involved in the biosynthesis of mycobactin, with three of these genes (*mbtL*, *mbtN*, and *mbtJ*) being absent in the *M. seoulense* strains relative to the MTB reference strain (H37Rv). The synthesis of the core mycobactin scaffold is mediated by enzymes encoded by *mbt* A-J, while the transfer of the lipophilic aliphatic chain to the ϵ-amino group of the lysine fragment is facilitated by enzymes encoded by *mbt*K-N, resulting in the potential loss of function of mycobactin in *M. seoulense* (Shyam et al., 2021). In addition, *faD33* (*mbtM*) was absent in *M. seoulense*, resulting in the absence of inhibitory effects by the antituberculosis agents (C16-AMS nucleoside) targeting *mbtM* (Guillet et al., 2016; Shyam et al., 2021). Moreover, *pks2* and *fad23* were absent in *M. seoulense*, which would impair the biosynthesis of Sulfolipid-1 (Yan et al., 2023). This protein can restrict the intracellular survival of MTB in human macrophages in a species-specific manner (Gilmore et al., 2012), and it can also serve as a nociceptive molecule that activates nociceptive neurons and induces coughing in infected animal models (Ruhl et al., 2020). The *rmlA* gene, which was not detected in the genome of *M. seoulense*, has been identified as a virulence factor enhancing the intracellular growth of MTB (Sassetti et al., 2003) and may be a target for antituberculosis agents (Xiao et al., 2021). Eis, a secreted protein not present in *M. seoulense* but present in other mycobacterial species, suppresses host innate immune defenses by modulating autophagy, inflammation, and cell death in a redox-dependent manner (Shin et al., 2010). *M. seoulense* strains lacking certain genes mentioned above were able to survive within macrophages, other virulence factors may have played a compensatory role in enhancing intracellular survival, such as those associated with the ESX-1 secretion system (Guo et al., 2021; Bo et al., 2023). Notably, *katA* is uniquely present in *M. seoulense*, while *KatG* is conserved across all the mycobacterial species (**Supplementary Table 3**). Both *katA* and *katG* encode catalase enzymes that are essential for detoxifying antimicrobial hydrogen peroxide (Stacy et al., 2014; Santander et al., 2018), with *katG* also contributing to isoniazid resistance (Ofori-Anyinam et al., 2024).

This study has several limitations. While certain virulence factor-encoding genes were uniquely detected or absent in the genomes of these *M. seoulense* strains, their precise roles in intracellular survival and pathogenicity remain unclear. The current *in vitro* macrophage infection data serve as an initial reference, but future studies utilizing gene-editing techniques will be necessary to elucidate their functional significance. Additionally, information regarding possible interactions between cases could not be obtained owing to the prolonged period over which these clinical isolates were obtained, although their living addresses were not geographically linked. Furthermore, clinical follow-up after species identification was not conducted, resulting in missing data on patient prognosis following *M. seoulense* infection.

## Conclusion

Based on the MICs measured, *M. seoulense* strains can be efficiently inhibited by rifampicin, rifabutin, linezolid, and clarithromycin but not by doxycycline, minocycline, or ciprofloxacin. Pan-genomic analyses revealed the core genes across all the strains, and the distribution of virulence factor-related genes differed from those of other common NTM species. Our findings may not only help clinicians select appropriate drug therapies for *M. seoulense*-related infections but may also provide possible targets for studies of the mechanisms underlying the pathogenicity of these mycobacteria.

## Data Availability

All the genomic sequences could be accessible from NCBI data with No. PRJNA1196008 and No. PRJNA1195676.

## Supplementary material

Supplementary Figure 1 The distribution of average nucleotide identity among *Mycobacterium seoulense* strains.
