# Supplementary figures and images for: Resistance phenotypes and genomic features of Mycobacterium seoulense isolates
Source: Front Cell Infect Microbiol. 2025 Apr 7;15:1553591. doi: 10.3389/fcimb.2025.1553591 (PMC12009822; doi:10.3389/fcimb.2025.1553591)

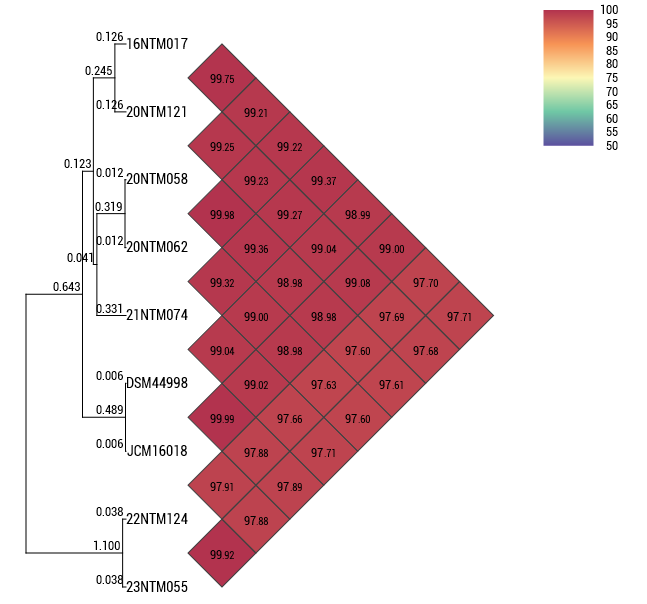

Supplement: Supplementary Figure 1 — The distribution of average nucleotide identity among Mycobacterium seoulense strains. [file Image1.tif]
