# Supplementary material for: Resistance phenotypes and genomic features of Mycobacterium seoulense isolates
Source: Front Cell Infect Microbiol. 2025 Apr 7;15:1553591. doi: 10.3389/fcimb.2025.1553591 (PMC12009822; doi:10.3389/fcimb.2025.1553591)
Supplement: Supplementary file 2 [file Table1.docx]

Supplementary Table 1. Clinical manifestation and laboratory examinations of patients with *M*. *seoulense*.

| Features | 20NTM062  20NTM058 | 20NTM121 | 21NTM074 | 16NTM017 | 22NTM124 | 23NTM055 |
| --- | --- | --- | --- | --- | --- | --- |
| Gender | Female | Male | Female | Female | Male | Female |
| Age (year) | 65 | 76 | 52 | 80 | 70 | 63 |
| Duration of  Symptoms (year) | >10 | >10 | >1 | >10 | >10 | No |
| TB history | No | No | No | Yes | No | No |
| Sample source | BAL | sputum | sputum | BAL | BAL | BAL |
| Positive/total events in Acid-fast | 0/7 | 0/16 | 0/1 | 1/12 | 0/4 | 0/2 |
| GeneXpert | Negative |  | Negative |  | Negative | Negative |
| T-Spot | Negative |  | Negative |  | Negative | Positive |
| Coexisting disease | Helicobacter pylori infection | AE-COPD, RF2, HBP2,  PH, TN, CSLD, GS, | sinus bradycardia,  CAP | bronchiectasis | AE-COPD, RF2, CPHD, CF3, BA, MPH, PH, LLA | SH, PTMC |

BAL, bronchoalveolar lavage fluid; PH, prostate hyperplasia; AE-COPD, acute exacerbation of chronic obstructive pulmonary disease; HBP2, 2 stage hypertension; RF2, type 2 respiratory failure; TN, thyroid nodule; CSLD, chronic schistosomiasis liver disease; GS, gallstone; CAP, community-acquired pneumonia; CPHD, chronic pulmonary heart disease; CF3, grade 3 cardiac function; BA, bronchial asthma; MPH, moderate pulmonary hypertension; LLA, lower limb atherosclerosis; SH, secondary hypothyroidism; PTMC, papillary thyroid microcarcinoma.
